# Use of human lymphocyte G0 PCCs to detect intra- and inter-chromosomal aberrations for early radiation biodosimetry and retrospective assessment of radiation-induced effects

**DOI:** 10.1371/journal.pone.0216081

**Published:** 2019-05-06

**Authors:** Terri L. Ryan, Antonio G. Pantelias, Georgia I. Terzoudi, Gabriel E. Pantelias, Adayabalam S. Balajee

**Affiliations:** 1 Cytogenetic Biodosimetry Laboratory, Radiation Emergency Assistance Center/Training site, Oak Ridge Institute for Science and Education, Oak Ridge Associated Universities, Oak Ridge, Tennessee, United States of America; 2 Health Physics, Radiobiology & Cytogenetics Laboratory, Institute of Nuclear & Radiological Sciences & Technology, Energy & Safety, National Centre for Scientific Research “Demokritos”, Ag. Paraskevi, Athens, Greece; ENEA Centro Ricerche Casaccia, ITALY

## Abstract

A sensitive biodosimetry tool is required for rapid individualized dose estimation and risk assessment in the case of radiological or nuclear mass casualty scenarios to prioritize exposed humans for immediate medical countermeasures to reduce radiation related injuries or morbidity risks. Unlike the conventional Dicentric Chromosome Assay (DCA), which takes about 3–4 days for radiation dose estimation, cell fusion mediated Premature Chromosome Condensation (PCC) technique in G0 lymphocytes can be rapidly performed for radiation dose assessment within 6–8 hrs of sample receipt by alleviating the need for *ex vivo* lymphocyte proliferation for 48 hrs. Despite this advantage, the PCC technique has not yet been fully exploited for radiation biodosimetry. Realizing the advantage of G0 PCC technique that can be instantaneously applied to unstimulated lymphocytes, we evaluated the utility of G0 PCC technique in detecting ionizing radiation (IR) induced stable and unstable chromosomal aberrations for biodosimetry purposes. Our study demonstrates that PCC coupled with mFISH and mBAND techniques can efficiently detect both numerical and structural chromosome aberrations at the intra- and inter-chromosomal levels in unstimulated T- and B-lymphocytes. Collectively, we demonstrate that the G0 PCC technique has the potential for development as a biodosimetry tool for detecting unstable chromosome aberrations (chromosome fragments and dicentric chromosomes) for early radiation dose estimation and stable chromosome exchange events (translocations) for retrospective monitoring of individualized health risks in unstimulated lymphocytes.

## Introduction

Exposure to ionizing radiation (IR) can adversely affect human health including mortality from acute radiation syndrome with a LD50/30 value of 3.5 Gy-4.5 Gy without treatment and 6.5 Gy- 7.5 Gy with appropriate therapy. Therefore, it is imperative to determine the absorbed radiation dose to initiate appropriate medical countermeasures. Bender and Gooch (1962) reported for the first time that the detection of dicentric chromosomes (DCs) in peripheral blood lymphocytes can be reflective of the absorbed radiation dose in exposed humans. Since then, Dicentric Chromosome Assay (DCA) has been routinely used for radiation dose assessment of either occupationally or accidentally exposed humans. DCA was effectively utilized in the past for radiation dose assessment in the victims of many well-known accidents such as Chernobyl [1–3], Goiania [4–6] and Fukushima-Daiichi [7–9]. The conventional DCA requires the stimulation of T-lymphocytes *in vitro* by Phytohaemagglutinin-M (PHA-M) for at least 48 hrs with an additional time of 24–48 hrs for cell fixation, DC analysis and radiation dose estimation. Although DCA is considered as the “gold standard” for radiation dose assessment, its labor intensive and time-consuming nature make DCA largely unsuitable for mass casualty incidents. Rapid individualized dose assessment is an absolute requirement for segregating people with moderate or high radiation exposure from non-exposed but “worried- well” population so that individuals who need urgent care can be prioritized for treatment.

Several efforts have been continually made to reduce the turnaround time for DCA: (I) optimization of chromosome preparation [10], (II) automated dicentric chromosome scoring [11–18], (III) sample tracking [19], (IV) establishment of network for cytogenetic biodosimetry laboratories [20–22], (V) data generation through electronic scoring of digital images [23–26] and a triage mode of scoring either 50 cells or 30 dicentrics [27–29]. Although these efforts significantly reduce the turnaround time for dose estimation, *in vitro* culturing of lymphocytes for 48 hrs is still inevitable for performing the conventional DCA. Further, sensitivity of lymphocytes to high doses of IR is yet another confounding factor that restricts the use of DCA in certain situations where radiation exposure exceeds 5 Gy. To overcome the technical limitations of DCA, Prematurely Condensed Chromosome (PCC) technique [fusion of non-stimulated G0 lymphocytes with Chinese Hamster Ovary (CHO) mitotic cells; hereafter referred as G0 PCC] was used for analyzing IR induced chromosomal aberrations [30–32]. The main advantage of using the PCC technique is that it enables chromosome aberration analysis instantaneously by alleviating the need for lymphocyte stimulation *in vitro* for 48 hrs. The PCC technique was effectively used in an earlier study for estimating the radiation dose from the frequency of excess chromosome fragments and rings [32]. The PCC technique was also utilized for detecting partial and whole body exposure of non-human primates [33]. One drawback of the PCC technique is that the centromeric regions are not readily detectable by conventional Giemsa staining technique. Initially, Pantelias et al. [34] used the centromeric heterochromatin banding technique (C-banding) for analyzing the frequency of IR induced dicentric chromosomes. Subsequently, the fluorescence *in situ* hybridization (FISH) technique was employed using DNA probes specific for human centromeres and telomeres for DC detection in PCCs obtained from non-stimulated lymphocytes [35–37]. Karachristou et al. [35] demonstrated the utility of PCC-FISH technique for triage biodosimetry by constructing a dose response curve up to 10Gy of γ-rays. An inter-laboratory comparison study on PCCs was recently undertaken within the RENEB (Realizing the European NEtwork of Biodosimetry) network of laboratories in Europe to improve the harmonization, standardization and optimization of the PCC assay for biological dosimetry [38].

The PCC technique is distinctly advantageous over the conventional DCA because it can be performed instantaneously after blood collection without the need for lymphocyte stimulation. In contrast to conventional DCA, which is routinely applied to the analysis of T-lymphocytes, PCC technique offers the flexibility of assessing radiation induced chromosomal aberrations in both T- and B-lymphocytes simultaneously. When lymphocytes are exposed to high radiation doses (> 5Gy), many heavily damaged lymphocytes may either die or may not even reach mitosis resulting in erroneous dose estimation when assayed by the conventional DCA. As the PCC assay does not involve *in vitro* lymphocyte proliferation, high radiation exposure exceeding 5 Gy is not a constraint for chromosome aberration analysis. Despite these advantages, the PCC technique is not quite widely used for biodosimetry. In the current study, we have evaluated and expanded the applicability of G0 PCCs for detecting different types of IR induced inter-chromosomal and intra-chromosomal aberrations by multicolor FISH (mFISH) and multicolor band (mBAND) techniques. Our study indicates the potential of using the PCC-FISH technique in detecting a wide variety of stable and unstable chromosomal aberrations for early dose estimation and retrospective assessment of radiation effects.

## Materials and methods

### Collection and irradiation of human blood samples

The study was performed with the written consent of human volunteers in compliance with the Oak Ridge Site Wide IRB (#ORAU 000379). ORISE/DOE granted the IRB approval. Peripheral blood samples in heparinized tubes were obtained from healthy male and female donors. Irradiation of whole blood samples was carried out *in vitro* using a Co-60 Gamma Cell 220 irradiator (Atomic Energy of Canada Ltd., Ottawa, Canada; 220 kV) at room temperature and at a dose rate of 0.2 Gy/min. Blood samples were exposed for different times to deliver doses ranging from 1 to 6 Gy. Following irradiation, blood samples were either processed immediately for lymphocyte isolation and their fusion for PCC induction (a procedure that allows approximately a repair time of 2 hrs), or allowed to repair for 6 hrs at 37°C before being processed for cell fusion and PCC induction. Sometimes, male and female lymphocytes isolated after irradiation were mixed for cell fusion and PCC generation. X-rays irradiation (RS 2000; RAD Source, 0.3mm copper filter that allows 160kV operation at 25mA; dose rate 2 Gy/min) of whole blood samples was performed using the X-ray irradiator at the University of Tennessee Knoxville, TN, USA. G0 PCCs after X-rays exposure were prepared at 2 hrs of post-recovery time at the Cytogenetic Biodosimetry Laboratory (CBL), Oak Ridge, TN.

### Preparation of the PCC-inducer mitotic CHO cells

Chinese hamster Ovary (CHO) cells were grown in McCoy’s 5A (Biochrom) medium supplemented with 10% FBS, 1% L-glutamine and 1% antibiotics (Penicillin, Streptomycin) at 37 °C in a humidified atmosphere with 5% CO2. CHO cells were maintained as exponentially growing monolayer cultures in 75 cm^2^ plastic flasks at an initial density of 4 × 10^5^ cells/flask. For optimizing the harvest of mitotic cells via cell synchronization, cells were allowed to grow until confluence and sub-cultured equally into three new 75 cm^2^ plastic flasks. Following 24–30 h of incubation at 37 °C, Colcemid (GIBCO, Life Technologies Corporation, Grand Island, NY) at a final concentration of 0.1 μg/ml was added to CHO cultures for 4 h and the mitotic cells were harvested by selective detachment. Once a sufficient number of mitotic cells had been obtained, they were used as supplier of mitosis promoting factors (MPF) to induce PCC in human G0 lymphocytes Alternatively, the harvested mitotic CHO cells can always be stored as frozen stocks to be used for PCC induction in G0 lymphocytes whenever needed.

### Cell fusion-mediated induction of premature chromosome condensation in lymphocytes

Lymphocytes from control and irradiated cells were isolated from whole blood using Biocoll separating solution (Biochrom, Berlin, Germany). The blood samples diluted 1:2 in RPMI-1640 without FBS were carefully layered on top of equal amounts of Biocoll in 12 ml test tubes and centrifuged at 400x g for 20 min. Collected lymphocytes from each tube were washed with 10 ml of culture medium (RPMI-1640 supplemented with 10% FBS, 1% glutamine and antibiotics), centrifuged at 300x g for 10 min and kept in culture medium before fusing them with mitotic CHO cells. The mitotic CHO cells harvested from a 75-cm2 flask were used for 2–3 fusions using the lymphocytes isolated from 1ml of whole blood sample for each experimental point.

Cell fusion and PCC induction were performed using 45% polyethylene glycol (PEG, p5402 Sigma–Aldrich, St. Louis, MO) in serum-free RPMI-1640 medium with HEPES. Lymphocytes and mitotic CHO cells were mixed in serum-free RPMI-1640 medium in a 12 ml round-bottom culture tube in the presence of colcemid. After centrifugation at 1000 rpm for 8 min, the supernatant was discarded without disturbing the cell pellet, keeping the tubes always inverted in a test tube rack on a paper towel to drain the pellet from excess liquid. While holding the tubes in an inverted position, 0.15 ml of PEG was injected forcefully against the cell pellet using a micropipette, and immediately after the tube was turned in an upright position and held for about 1 min. Subsequently, 1.5 ml of PBS was slowly added to the tube with gentle shaking and the cell suspension was centrifuged at 1000 rpm for 8 min. The supernatant was discarded and the cell pellet was suspended gently in 0.7 ml RPMI-1640 complete growth medium with HEPES containing PHA and colcemid. The tubes were incubated for 75 min at 37°C for the completion of cell fusion/PCC induction. Cells were then treated with hypotonic KCl (0.075 M) and fixed with two changes of methanol: glacial acetic acid (v/v 3:1). The chromosome spreads were prepared by the standard air-drying technique and slides were stained using 3% Giemsa in Sorensen buffer solution for PCC analysis. G0 lymphocyte PCCs appear as single chromatid chromosomes and can be easily distinguished from CHO metaphase chromosomes, which have two chromatids per chromosome. Alternately, the slides were subjected to fluorescence *in situ* hybridization (FISH) for detecting both unstable (dicentric chromosomes) and stable (translocations and inversions) chromosomal aberrations induced by IR. Cell fusion after X-rays exposure was performed at the CBL, Oak Ridge using both PEG and Sendai virus (HVJ) Envelope Cell Fusion Kit (Cosmo Bio, Carlsbad, CA) following the manufacturer’s instructions.

### Fluorescence *in situ* hybridization (FISH), multicolor FISH (mFISH) and multicolor BAND (mBAND)

Procedure for the fluorescence *in situ* hybridization (FISH) technique using peptide nucleic acid (PNA) based human telomeric and centromeric DNA probes was essentially the same as described in our previous studies [39, 40]. A cocktail of probe specific for human chromosomes 1, 2 and 4 was obtained from MetaSystems and the FISH procedure using this probe was performed essentially as described by the manufacturers. The mFISH technique was performed essentially as described before [39]. Briefly, slides were treated for 1 min with 0.001% acidic pepsin solution (0.01NHCl) at 37°C for 1–2 min followed by two washes of 5 min each in phosphate-buffered saline. The slides were post-fixed for 10 min in a solution of formaldehyde/MgCl_2_ (1% formaldehyde/50mM MgCl_2_ in PBS). The slides after denaturation (2X SSC at 70°C for 20 min and after cooling to ambient temperature 1 min in 0.07N NaOH) were dehydrated in graded series of ethanol (30%, 70%, 90% and 100%) and air dried. The mBAND probe was denatured separately by incubation at 75°C for 5 min followed by incubation at 37°C for 30 min to allow the annealing of repetitive DNA sequences. An aliquot of 10μl probe was placed on the slide and covered with a coverslip. The slides were kept in a humidified hybridization chamber at 37°C for at least 72hr. The unbound probe was removed by washing the slides in pre-warmed (75°C) 1X SSC (pH 7.0–7.5) for 5 min followed by incubation in 4XSSCT (4X SSC with 0.1% Tween 20) for 5 min. Indirectly labeled probe (Cy5), if needed, was amplified by incubation with antibodies (biotinylated anti-streptavidin and Cy5 conjugated streptavidin; Invitrogen, Carlsbad, CA, USA) sequentially for 30 min followed by two washes of 3 min each in 4XSSCT and in PBS. The nuclei were counterstained with DAPI (Vectashield Laboratories, Burlingame, CA, USA). Images were captured using the Zeiss epifluorescence microscope. Image analysis was performed using the ISIS software (MetaSystems, Boston, MA, USA) essentially according to the published procedure. Normal and aberrant chromosomes were identified by unique chromosome specific processed color generated by the ISIS software based on pixel intensities of combinatorial labeling of the five fluorochromes (FITC, Spectrum Orange, Texas Red, DEAC and Cy5). Human chromosome 5 specific multicolor BAND was obtained from MetaSystems. Procedures for hybridization, post-hybridization washes and detection were essentially the same as described for mFISH. Intra-chromosomal aberrations were scored using the ISIS software. Fluorescently labeled gene specific probes sets were purchased from CytoCell, Lincolnshire, IL, USA. The frequencies of chromosomal aberrations are expressed as mean with lower and upper confidence limits at 95% interval.

## Results

The G0 PCC-FISH technique is distinctly advantageous because the turnaround time for radiation dose assessment is dramatically reduced (6–8 hrs after the receipt of blood samples relative to 72–96 hrs by the conventional DCA). Previous studies on G0 PCC have focused mainly on dicentric chromosomes for radiation dose assessment [34, 35, 41]. The current study was undertaken to determine the applicability of PCC-FISH technique for detecting both inter- and intra-chromosomal aberrations using a wide variety of multicolor DNA probes.

### Detection of dicentric chromosomes by centromere/telomere FISH after γ-rays exposure

As stated before, centromeric regions are not clearly detectable in G0 PCCs when conventional Giemsa staining technique is used. Therefore, the frequency of dicentric chromosomes was analyzed in the present study by centromere and telomere FISH in G0 PCCs obtained at 6 hrs after exposure to varying doses of γ-rays (0 Gy, 1 Gy, 2 Gy, 4 Gy and 6 Gy). The frequency as well as the distribution of dicentric chromosomes observed in 50 fused cells for each radiation dose is summarized in Table 1. In corroboration with earlier studies, a dose dependent increase in dicentric chromosomes was observed (Mean ± SEM; 0.06 ± 0.03/cell for 1 Gy, 0.14 ± 0.05/cell for 2 Gy, 1.32 ± 0.24 /cell for 4 Gy and 2.48 ± 0.41/cell for 6 Gy) in the PCC spreads. Consistent with the increase in dicentric chromosome number, the total number of chromosome objects (fragments) also increased as a function of radiation dose presumably owing to excess fragments resulting from chromosome breakage. At the highest dose of 6 Gy, the number of excess fragments ranged from 2–12 with mean value of 6.60. Earlier studies [35, 42] have demonstrated that the initial yield of fragments was high at 2 hrs after radiation exposure but reached a plateau at 6–8 hrs post-exposure. We compared γ-rays induced dicentric frequencies between G0 PCC and conventional DCA by the triage mode of scoring (50 cells or 30 dicentrics) and found that the frequencies detected by the two assays were grossly similar for different radiation doses: G0 PCC (0.06/cell for 1 Gy, 0.14/cell for 2 Gy, 1.32/cell for 4 Gy and 2.48/cell for 6 Gy) and conventional DCA (0.06/ cell for 1 Gy, 0.38/cell for 2 Gy, 1.42/cell for 4 Gy and 2/cell for 6 Gy). It is interesting to note that the dicentric frequency detected by the conventional DCA for 6 Gy was less than that detected by G0 PCC probably owing to either increased death of severely damaged cells or inability of severely damaged cells to progress to mitosis due to a prolonged cell cycle arrest at G2.

**Table 1:** Detection of dicentric chromosomes in γ-rays irradiated human G0 PCCs by centromere and telomere FISH.

Dose (Gy)	Cells scored	Distribution of dicentrics							Mean	95% CI
		**0**	**1**	**2**	**3**	**4**	**5**	**>5**		
0	50	50	0	0	0	0	0	0	0.00	0.00
1	50	47	3	0	0	0	0	0	0.06	0.01–0.11
2	50	45	3	2	0	0	0	0	0.14	0.05–0.23
4	50	12	20	10	6	2	0	0	1.32	0.85–1.79
6	50	6	8	10	14	8	3	1	2.48	1.68–3.28

### Detection of inter-chromosomal exchange events by multicolor FISH after γ-rays exposure

**Fig 1:**
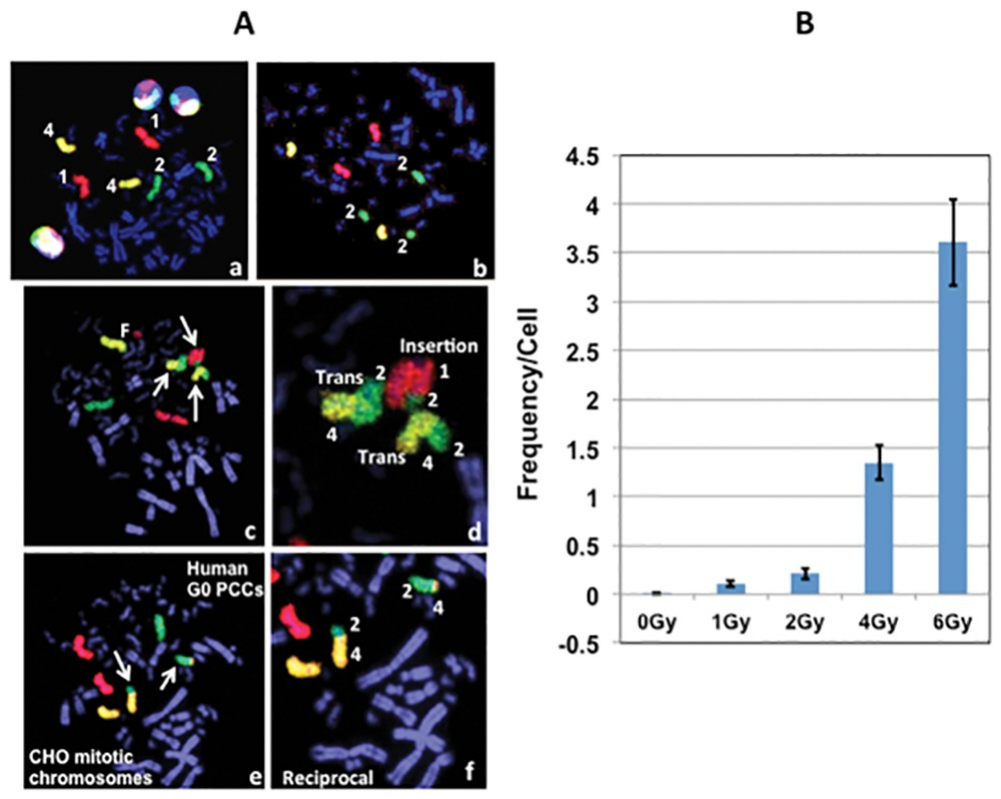
(A) Detection of inter-chromosomal exchange events in human G0 PCCs prepared 6 hrs after exposure to different doses of γ-rays. (a) A cocktail probe specific for chromosomes 1 (red color), 2 (green color) and 4 (yellow color) was used for detection. (b) Detection of chromosome 2 fragment. (c) Detection of reciprocal translocation between painted chromosomes 2 and 4 and an insertion of chromosome 2 on chromosome 1 (arrows). (d) Magnification of the same cell showing reciprocal translocation and insertion. (e) Reciprocal translocation detected between chromosomes 2 and 4 (arrows). (f) Magnification of the same cell shown in e. (B) Frequency of inter-chromosomal exchange events observed at different radiation doses of γ-rays. Mean ± SEM.

Although dicentric chromosome detection has been well established in G0 PCCs, no attempt has been made to determine the feasibility of using G0 PCCs for detecting stable/symmetrical chromosomal aberrations. We evaluated this feasibility by using a cocktail of fluorescently labeled DNA probes specific for human chromosomes 1 (Texas Red), 2 (fluorescein) and 4 (Both Texas Red and fluorescein yielding a yellow color). For painting, PCCs prepared at 6 hrs of post-recovery from lymphocytes irradiated with different doses of γ-rays (0 Gy, 1 Gy, 2 Gy, 4 Gy and 6 Gy) were utilized. Color junctions, which are reflective of inter-chromosomal exchange events involving painted and non-painted chromosomes, were scored in 60–100 cells for each radiation dose (Table 2). Representative images with reciprocal translocation and insertion detected by whole chromosome specific cocktail probe are shown in Fig 1A. The frequencies of color junctions observed for various doses of γ-rays are shown in Fig 1B. The frequency of inter-chromosomal exchange events showed a dose dependent increase in PCCs prepared after 6 hrs of recovery time (0.01 for 0 Gy, 0.11 for 1 Gy, 0.21 for 2 Gy, 1.35 for 4 Gy and 3.61 for 6 Gy).

**Table 2:** Detection of γ-rays induced inter-chromosome exchange events in prematurely condensed G0 human chromosomes using whole chromosome specific DNA cocktail probe (Chr. 1, 2 and 4).

Dose (Gy)	Cells scored	Distribution of exchanges							Mean	95% CI
		**0**	**1**	**2**	**3**	**4**	**5**	**>5**		
0	100	99	1	0	0	0	0	0	0.01	-0.01–0.03
1	100	93	3	4	0	0	0	0	0.11	0.06–0.16
2	67	58	5	3	1	0	0	0	0.21	0.09–0.33
4	100	36	26	20	8	7	2	1	1.35	1.02–1.68
6	85	12	10	13	9	9	8	24	3.61	2.75–4.47

**Fig 2:**
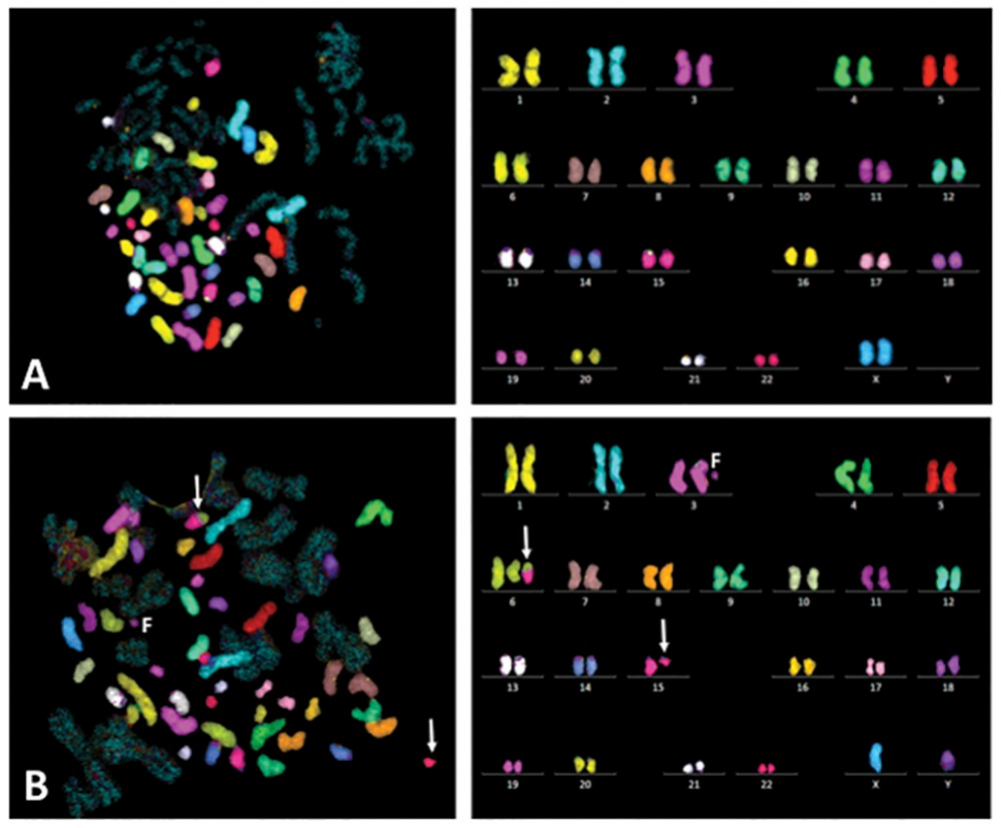
Multicolor FISH (mFISH) hybridization of human G0 PCCs. (A) Normal lymphocyte PCC spread and mFISH karyotype. (B) Abnormal lymphocyte PCC spread (G0 PCCs prepared 6 hrs after 1 Gy of γ-rays exposure) and mFISH karyotype showing a translocation t(6:15) and a fragment of chromosome 3. Arrows-chromosomes involved in translocation.

**Fig 3:**
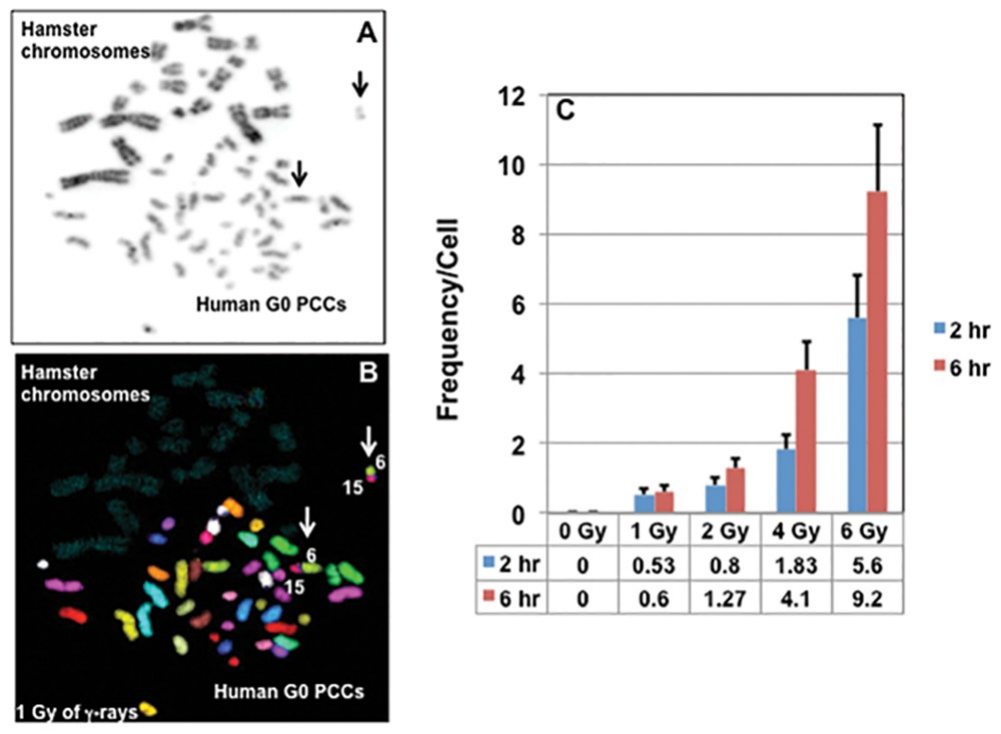
Detection of translocations in human G0 PCCs by mFISH. Note the reciprocal translocation involving chromosomes 6 and 15 (arrows) in G0 PCCs prepared from 1 Gy γ-rays treated cells at 6 hrs of post-recovery. (A) DAPI counter stained cell shown in grey scale (B) mFISH hybridization pattern of the same cell with a reciprocal translocation involving chromosomes 6 and 15. (C) Frequencies of inter-chromosomal exchanges observed for different doses of γ-rays at different post-recovery times. Mean ± SEM. Arrows-translocated chromosomes.

Using the mFISH technique, genome wide analysis was performed for the first time in G0 PCCs to detect IR induced simple and complex chromosome exchange events (translocations). As each of the homologous chromosome pair is color coded, simple and complex translocations can be easily detected. Color junctions, which are reflective of inter-chromosomal exchange events, were scored in G0 PCCs prepared at 2hrs (Table 3) and 6 hrs (Table 4) after exposure to various doses of γ-rays (0 Gy, 1 Gy, 2 Gy, 4 Gy and 6 Gy). Representative images of mFISH karyotypes prepared from mock and 1 Gy γ-rays treated G0 PCCs are shown in Fig 2. The frequencies of chromosome exchanges observed in G0 PCCs at 2 hrs and 6 hrs post-radiation exposure times are given in Fig 3 and Table 2. The frequency of color junctions detected in G0 PCCs did not differ significantly between 2 hrs and 6 hrs for 1 Gy and 2 Gy of γ-rays exposure. However, number of color junctions dramatically increased at 6 hrs relative to 2 hrs for both 4 Gy and 6 Gy doses. The frequency of color junctions observed in G0 PCCs prepared after 2 hrs of γ-rays exposure was 0.53/cell for 1 Gy, 0.80/cell for 2 Gy, 1.83/cell for 4 Gy and 5.60/cell for 6 Gy while the frequency of color junctions detected in G0 PCCs after 6 hrs of exposure was 0.60/cell for 1 Gy, 1.27/cell for 2 Gy, 4.10/cell for 4 Gy and 9.20/cell for 6 Gy.

**Table 3:** Multicolor FISH detection of γ-rays induced inter-chromosome exchange events in prematurely condensed G0 human chromosomes prepared 2 hrs after exposure.

Dose (Gy)	Cells scored	Distribution of exchanges							Mean	95% CI
		**0**	**1**	**2**	**3**	**4**	**5**	**>5**		
0	30	30	0	0	0	0	0	0	0.00	0.00
1	30	20	5	4	1	0	0	0	0.53	0.22–0.84
2	30	18	5	4	1	2	0	0	0.80	0.39–1.21
4	30	9	5	7	4	2	2	1	1.83	1.03–2.63
6	25	0	0	0	1	4	11	9	5.60	3.23–7.97

**Table 4:** Multicolor FISH detection of γ-rays induced inter-chromosome exchange events in prematurely condensed G0 human chromosomes prepared 6 hrs after exposure.

Dose (Gy)	Cells scored	Distribution of exchanges							Mean	95% CI
		**0**	**1**	**2**	**3**	**4**	**5**	**>5**		
0	30	30	0	0	0	0	0	0	0.00	0.00
1	30	19	5	5	1	0	0	0	0.60	0.25–0.95
2	30	16	1	8	1	3	0	1	1.27	0.67–1.87
4	30	2	8	3	2	1	2	12	4.10	2.48–5.72
6	25	0	0	0	1	1	1	22	9.20	5.42–12.98

It is of interest to note that the average number of chromosome fragments in G0 PCCs showed a decline at 6 hrs for all the radiation doses (46.70/cell for 1 Gy, 47.80/cell for 2 Gy, 49.20/cell for 4 Gy and 54.40/cell for 6 Gy) relative to PCCs prepared at 2 hrs post exposure (47.90/cell for 1Gy, 50.40/cell for 2 Gy, 61/cell for 4 Gy and 68.60/cell for 6 Gy). The decline in the number of painted objects observed in the PCCs at 6 hrs after exposure is probably due to rejoining/mis-rejoining of some of the excess fragments. Similar to the induction of dicentric chromosomes, a dose dependent increase in the number of color junctions was observed by the mFISH technique.

**Fig 4:**
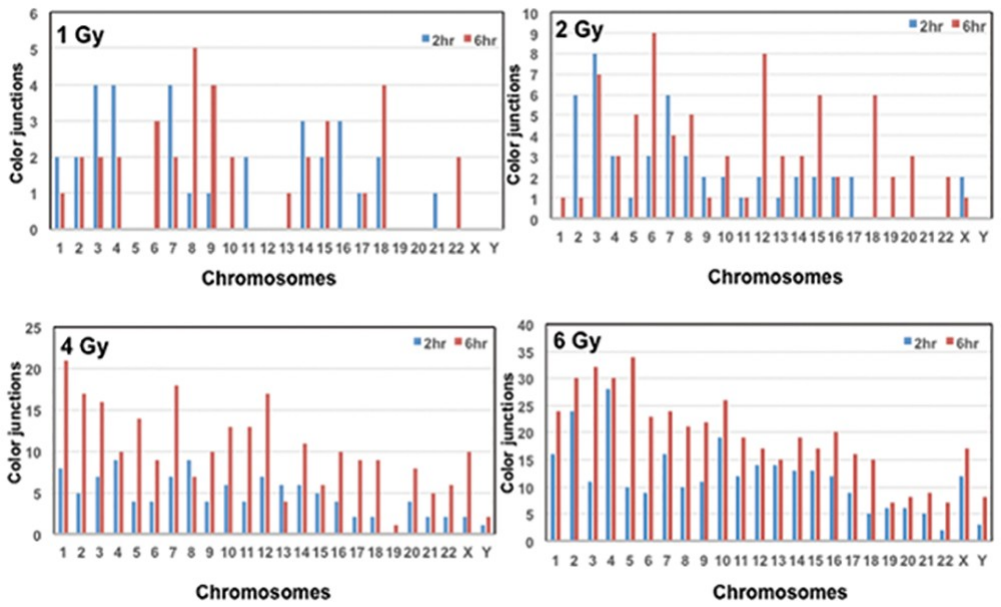
Chromosome specific distribution of color junctions detected by mFISH technique in human G0 PCCs after exposure to varying doses of γ-rays exposure at different post-recovery times (2 hrs and 6 hrs).

We next determined whether or not IR induced exchange events detected by mFISH occurred randomly or in a chromosome specific manner. The chromosome wide distribution of color junctions observed in the PCCs prepared at 2 hrs and 6 hrs post-recovery are shown in Fig 4. In general, the number of color junctions observed for all the chromosomes was more for 6 hr PCCs than 2 hr PCCs without any preferential involvement of specific chromosomes. Interestingly, no color junctions were recorded for chromosomes 5, 12, 19, 20, X and Y for either of the post- recovery times after 1 Gy of γ-rays exposure. Likewise, chromosomes 21 and Y did not involve in any exchange events following 2 Gy exposure at both recovery times. Color junctions involving chromosomes 3, 5 and 6 showed a substantial increase at 6 hrs post recovery relative to 2 hrs after exposure to 6 Gy of γ-rays. The number of cells analyzed (typically 25–30) for each radiation dose and recovery time) was too low for any meaningful statistical analysis.

### Detection of inter-chromosomal exchange events by multicolor FISH after X-rays exposure

Analysis of X-rays induced inter-chromosomal events was performed on G0 PCCs using both whole chromosome specific cocktail and mFISH probes. Results obtained on the frequencies of exchanges are shown in Table 5. G0 PCCs were prepared from human lymphocytes 2 hrs after irradiation with different doses of X-rays (0 Gy, 2 Gy and 4 Gy). The frequency of exchanges detected using a cocktail probe specific for chromosomes 1, 2 and 4 was 0/cell for 0 Gy, 0.40/cell for 2 Gy and 0.85/cell for 4 Gy. As expected, genome wide distribution of inter-chromosomal exchanges detected by mFISH was higher for all the radiation doses (mean ± SE; 0.02 ± 0.02 for 0 Gy, 0.75 ± 0.21 for 2 Gy and 2.10 ± 0.48 for 4 Gy). The results of multicolor FISH for X-rays induced exchange events are shown in Table 6. Collectively, we demonstrate that G0 PCCs can be successfully utilized for detecting inter-chromosomal exchange events for rapid biodosimetry.

**Table 5:** Detection of X-rays induced inter-chromosome exchange events in prematurely condensed human G0 chromosomes using whole chromosome specific DNA cocktail probe (Chr. 1, 2 and 4).

Dose (Gy)	Cells scored	Distribution of exchanges				Mean	95% CI
		**0**	**1**	**2**	**3**		
0	100	100	0	0	0	0.00	0.00
2	65	46	13	5	1	0.40	0.23–0.57
4	60	31	11	12	6	0.85	0.54–1.16

**Table 6:** Detection of X-rays induced inter-chromosome exchange events in prematurely condensed G0 human chromosomes using multicolor FISH probe.

Dose (Gy)	Cells scored	Distribution of exchanges						Mean	95% CI
		**0**	**1**	**2**	**3**	**4**	**5**		
0	50	49	1	0	0	0	0	0.02	-0.01–0.05
2	28	21	1	2	1	2	1	0.75	0.34–1.16
4	28	8	2	3	10	4	1	2.10	1.16–3.04

### Detection of intra-chromosomal aberrations in G0 PCCs by mBAND after γ-rays exposure

**Fig 5:**
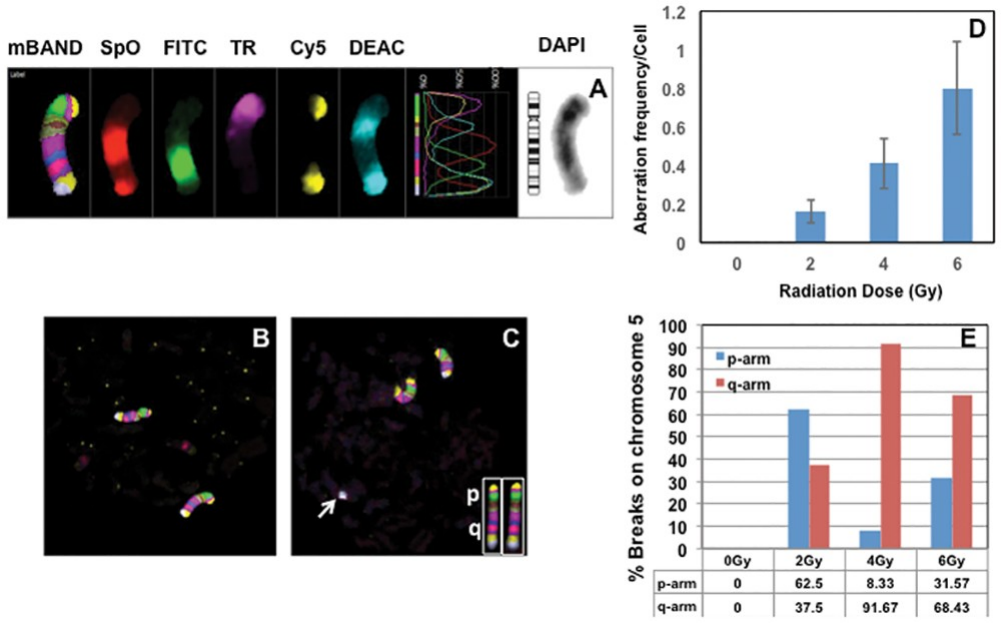
Detection of intra-chromosomal aberrations in G0 PCCs using chromosome specific mBAND probe. (A) The hybridization pattern of five different fluorochromes [SpO- Spectrum Orange, FITC-Fluorescein isothiocyanate, TR-Texas Red, Cy5- Cyanine 5 and DEAC-7-diethylaminocoumarin; DAPI (4′, 6-diamidino-2-phenylindole-chromosome counterstain]. Representative G0 PCCs of control (B) and irradiated (C; 4 Gy of γ-rays) lymphocytes probed with chromosome 5 specific mBAND probe are shown. Note the terminal fragment of one of the chromosomes 5 (arrow) in the irradiated G0 PCC spread. The hybridization patterns observed in the p- and q-arms of metaphase chromosome 5 are shown in the insert. (D) Frequency of total intra-chromosomal aberrations (fragments of p and q arms, translocations and inversions) detected for different γ-rays doses in G0 PCCs. (E) Frequency of breaks observed in the p- and q-arms of the chromosome 5 detected by the mBAND technique. The percentage of breaks observed in the short and long arms of chromosome 5 for different γ-rays doses is shown in the form of histogram. Bars represent SEM.

Suitability of G0 PCCs for detecting IR induced intra-chromosomal aberrations (inversions, insertions, interstitial and terminal fragments) was next evaluated using chromosome 5 specific mBAND probe. For the detection of intra-chromosomal changes, G0 PCCs prepared at 6 hrs of post-recovery were used. Despite the prematurely condensed nature of G0 chromosomes, mBAND probe resulted in a reasonably good resolution of bands to enable the detection of intra-chromosomal changes (Fig 5A–5C). The frequencies of total intra-chromosomal aberrations on chromosome 5 (chromosome fragments, translocations and inversions) resulting from breaks induced in the p- and q- arms by different doses of γ-rays were scored in a total of 25–50 cells (50 cells for 0 Gy, 2 Gy and 4 Gy and 25 cells for 6 Gy) and the results are summarized in Fig 5D. We detected 3 translocations in 4 Gy irradiated G0 PCCs and 1 inversion in 6 Gy irradiated G0 PCCs. Although not useful for biodosimetry, mBAND enables the mapping of IR induced chromosome breakage sites and this feature will be particularly useful to determine whether or not certain chromosomal sites are prone to IR induced breaks. A representative lymphocyte PCC spread with a terminal fragment of chromosome 5 is shown in Fig 5C. At 2 Gy of exposure, breaks in the p-arm were slightly more than the q-arm but at higher doses (4 Gy and 6 Gy) breaks in the q-arm were substantially higher than the p-arm (Fig 5E). Our study indicates the feasibility of using the mBAND technique for detecting IR induced intrachromosomal aberrations in G0 PCCs.

### Detection of specific genes on G0 PCCs

**Fig 6:**
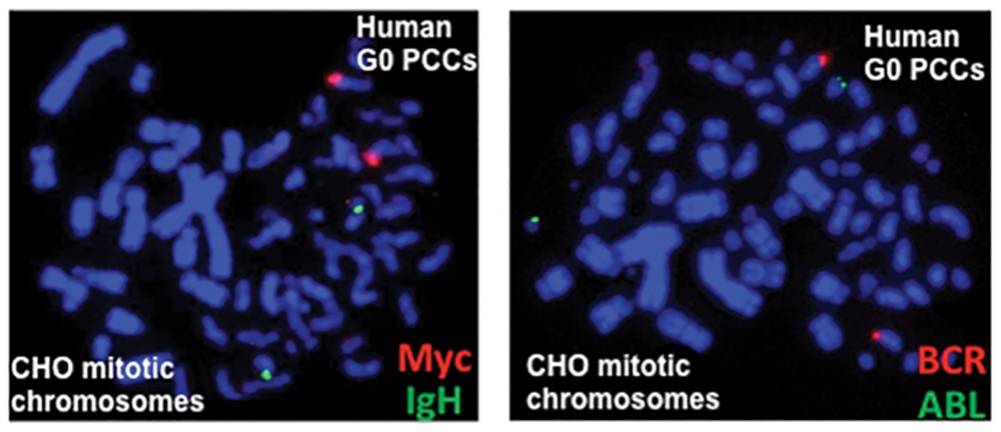
Detection of gene loci in G0 PCCs by FISH using gene specific probe sets. Fluorescently labeled gene probe sets (c-Myc-Texas Red and IgH- Fluorescein; BCR-Fluorescein and ABL-Texas Red) were used for detection. PCCs prepared from 3 Gy X-rays treated lymphocytes were used for detection.

We next evaluated the utility of G0 PCCs for detecting individual genes. G0 PCCs obtained from control and X-rays irradiated lymphocytes were probed with gene specific probe sets (c-Myc and IgH and BCR and ABL; Fig 6). Recently, we detected γ-rays induced translocation, amplification and gene fusions involving Myc and IgH genes in metaphase chromosomes (unpublished observation). Our demonstration of using gene locus specific FISH on G0 PCCs holds great promise for evaluating IR induced alterations involving either copy number changes or fusion/fission events at the gene specific level in the future.

## Discussion

DCA is considered to be the gold standard for radiation dose assessment but it requires proliferation of lymphocytes *in vitro* for 48 hrs to obtain metaphase chromosomes for analysis. Therefore, a turnaround time of 3–4 days is required at minimum for DCA to estimate an individual’s absorbed radiation dose. Further, it is somewhat difficult to perform conventional DCA for radiation doses higher than 5 Gy because lymphocytes, owing to their radiation sensitivity, may fail to proliferate or undergo apoptotic death at high radiation doses. Realizing the time consuming and laborious nature of DCA, an alternative method was developed by preparing prematurely condensed chromosomes in unstimulated human G0 lymphocytes to detect IR induced chromosomal aberrations. As the PCC assay bypasses the need for lymphocyte proliferation, chromosomal aberrations induced by a wide range of radiation exposures up to 20 Gy can be easily measured within 6–8 hrs of blood collection. PCC assay has been successfully utilized in earlier studies for analyzing IR induced dicentric chromosomes, rings and excess chromosome fragments [31, 32, 34, 43–45]. Recent studies have assessed the utility of G0 PCCs for triage biodosimetry by using the FISH technique with telomere and centromere specific PNA probes [35, 37, 41]. Based on the *ex vivo* data obtained, Lamadrid Boada et al., [42] suggested the use of PCC-rings for dose estimation in severely exposed humans. Interestingly, the frequency of rings remained essentially the same in *ex vivo* irradiated lymphocytes irrespective of the post-recovery times used for PCC preparation (8 hrs and 24 hrs). Recently, Pantelias and Terzoudi [46] developed an automatable micro-PCC assay for rapid individualized dose estimation during large-scale radiological emergencies. This technique will enhance the practical applicability of G0 PCC as an effective triage tool.

In some of the earlier studies, Calyculin A induced G2 PCC technique was used to detect IR induced chromosome translocations [47, 48] but this technique, unlike G0 PCCs, also involves *ex vivo* stimulation of human lymphocytes for 48 hrs. A major advantage of using the G2 PCC technique is that it yields sufficient number of G2 cells even at higher radiation doses (5–20 Gy) as most heavily damaged cells at these doses may not even progress to mitosis for conventional metaphase chromosome analysis. To date, cell fusion mediated G0 PCCs have been extensively used only for the analysis of asymmetrical or unstable chromosomal aberrations (dicentric chromosomes, rings and fragments). Our study is perhaps the first one to demonstrate the applicability of G0 PCCs for analyzing both inter- and intra-chromosomal aberrations using multicolor FISH and chromosome specific mBAND probes. Efficient detection of chromosomal translocations (simple and complex) by mFISH indicates a potential possibility of utilizing G0 PCCs for retrospective biodosimetry and for monitoring long-term effects of IR exposure on the hematopoietic system. The hybridization signal for either whole chromosome specific probe or mFISH probe was not affected by prematurely condensed nature of interphase chromosomes. As demonstrated in the current study, G0 PCCs can be easily karyotyped for numerical and structural analyses of aberrations induced by IR. The mFISH has an additional advantage over conventional Giemsa staining in identifying chromosome specific origin of excess fragments. In the current study, frequency of inter-chromosomal exchange events was compared by mFISH in G0 PCCs after 2 hrs and 6 hrs of exposure with different doses of γ-rays. Using a combination of centromere and telomere staining, an earlier study demonstrated that the rejoining of chromosome fragments reached a plateau at 8 hrs after radiation exposure (Karachristou et al., 2015). Previous studies [33, 49] suggested that exchange type aberrations predominantly arising from two lesions from a single ionizing track are formed instantly after low doses of X-rays (< 2Gy) while those exchange events resulting from two ionizing tracks at higher doses (> 2 Gy) increase as a function of post-recovery time. In corroboration, an increased yield of inter-chromosomal exchange events was detected by mFISH in G0 PCCs after 6 hrs of radiation exposure relative to 2 hrs, most notably for 4 Gy and 6 Gy doses of γ-rays.

In the current study, color junctions were scored for inter-chromosomal exchange events, which besides translocations (non-reciprocal and reciprocal exchanges) also included dicentric chromosomes. Using centromere and telomere FISH staining, frequency of dicentric chromosomes observed per cell was found to be 0.06 for 1 Gy, 0.14 for 2 Gy, 1.32 for 4 Gy and 2.48 for 6 Gy in G0 PCCs prepared after 6 hrs of post recovery. However, frequencies of color junctions detected by mFISH were much higher (0.6/cell for 1 Gy, 1.27/cell for 2 Gy, 4.1 for 4 Gy and 9.2 Gy for 6 Gy) than the dicentric frequencies observed by centromere and telomere staining. These observations indicate that the translocation frequency detected in G0 PCCs was higher than dicentric chromosome frequency although a direct comparison was not performed in our study to estimate the yields of dicentrics and translocations in the same cells. Using a pan centromeric and whole chromosome specific probes, Bauchinger et al. [50] detected more translocations than dicentrics. Although DNA double strand break is considered to be the critical lesion for the formation of exchange type aberrations (dicentrics and translocations), reports of dicentric to translocation ratios greater than 1 [50–53] suggest that the DSB rejoining processes may be different for their formation. In support, post-treatment of X-rays irradiated human lymphocytes with DNA repair inhibitors [Cytosine arabinoside (araC)–an inhibitor of DNA polymerase α; 3-aminobenzamide (3AB)—an inhibitor of Poly (ADP) Ribose Polymerase] potentiated only the formation of dicentric chromosomes but not translocations [52–56]. Strikingly, treatment of cells with araA (Adenine arabinofuranoside) specifically enhanced the frequencies of translocations but not dicentric chromosomes [53]. Although, we did not perform a direct comparison on the yield of radiation induced inter-chromosomal exchange events between interphase and metaphase chromosomes, chromatin structure and cell cycle stage may be important determinants for the formation of specific types of chromosome aberrations. In corroboration, differential yield of dicentrics and translocation was reported in Calyculin A induced G2 PCCs and colcemid arrested metaphase chromosomes in lymphocytes exposed to protons and carbon ions [57]. In the present study, distribution of color junctions detected by mFISH in G0 PCCs appeared to be largely random without involving any specific chromosomes. In general, the number of color junctions induced by 6 Gy of γ-rays in the G0 PCCs prepared 6 hrs after radiation exposure seemed to correlate with chromosome size. Muhlman-Diaz and Bedford [58] analyzed the breaks on chromosomes 4, 19 and Y in non-cycling human skin fibroblasts immediately after X-rays exposure and found no correlation between breakage per unit length and the ratio of AT/GC sequences for these chromosomes. The only exception was the euchromatic portion of the Y chromosome, which showed almost twice the number of chromosome breaks than expected.

In addition to inter-chromosomal exchanges, intra-chromosomal exchanges were also detected in G0 PCCs using chromosome 5 specific mBAND probe. The banding pattern observed in G0 PCCs was identical to colcemid arrested metaphase chromosomes despite the difference in chromosome size and chromatin condensation. In addition to chromosome breaks in the p and q-arms, translocation and inversion events were also detectable using the mBAND technique in G0 PCCs. Although not relevant for radiation biodosimetry, it is worth noting that the frequency of breaks detected in the q- arm was much higher than the p-arm at both radiation doses (4 Gy and 6 Gy) in G0 PCCs. Johannes et al. [59] reported similar observations in the metaphase chromosomes of human lymphocytes irradiated with 4 Gy of X-rays and the increased number of breaks observed in the q-arm correlated well with the DNA content (27% for the p-arm and 73% for the q-arm). Interestingly, we observed almost a 10 fold increase in breaks in the q-arm relative to p-arm after 4 Gy of X-rays. The number of cells analyzed was small to allow any meaningful statistical analysis for the distribution of breaks in the short and long arms of chromosome 5. Our study indicates that G0 PCCs can be successfully utilized for detecting intra-chromosomal exchange events such as inversions and translocations for retrospective dosimetry.

Although no evidence was provided in this study for gene fusion/fission events, successful demonstration of gene loci indicates the feasibility of detecting some of the radiation induced gene fusion/fission events in unstimulated lymphocytes. As gene fusion and fission events are important for cancer development processes, systematic analysis of these changes using G0 PCCs may yield valuable information for radiation induced carcinogenesis. Collectively, our study demonstrates that G0 PCCs can be used effectively for detecting a whole spectrum of inter- and intrachromosomal aberrations. As the cell fusion technique coupled with FISH can dramatically reduce the turnaround time for dose estimation by alleviating the need of *ex vivo* lymphocyte stimulation for 48 hours, we feel that the G0 PCC technique has the potential for future development as a biodosimetry tool: unstable chromosome aberrations (chromosome fragments and dicentric chromosomes) for early dose estimation and chromosome exchange events in stable cells for retrospective effects of radiation exposure in unstimulated lymphocytes without any bias or selection for only metaphase cells obtained after *ex vivo* stimulation.

## Supporting information

S1 Table Detection of dicentric chromosomes in γ-rays irradiated human lymphocyte G0 PCCs by centromere/telomere FISH. Distribution of dicentric chromosomes observed in a cell to cell basis and the range of total chromosome objected observed including excess fragments for different doses are shown.
(DOCX)

S2 Table Detection of γ-rays induced inter-chromosome exchange events detected by whole chromosome specific DNA cocktail probe (Chr.1, 2 and 4). Distribution of chromosome exchange events observed for varying radiation doses are shown.
(DOCX)

S3 Table Detection of γ-rays induced inter-chromosome exchange events detected by multicolor FISH in human G0 lymphocyte PCCs prepared 2 hrs after exposure. Cellular distribution of chromosome exchange events observed for different doses of γ-rays is shown.
(DOCX)

S4 Table Detection of γ-rays induced inter-chromosome exchange events detected by multicolor FISH in human G0 lymphocyte PCCs prepared 6 hrs after exposure. Cells with varying number of inter-chromosome exchange events (0–16) observed after exposure to varying radiation doses are shown.
(DOCX)

S5 Table Detection of X-rays induced inter-chromosome exchange events in prematurely condensed human chromosomes using whole chromosome specific DNA cocktail probe (Chr.1, 2 and 4). Cellular distribution of chromosome exchange events induced by X-rays is shown.
(DOCX)

S6 Table Detection of X-rays induced inter-chromosome exchange events in prematurely condensed human chromosomes using multicolor FISH. Cellular distribution of chromosome exchange events are shown for various doses of X-rays.
(DOCX)

S7 Table Analysis of γ-rays induced intra-chromosomal exchanges detected by the mBAND technique on chromosome 5. Data used for the generation of histogram plots presented in Fig 5D and 5E are shown.
(DOCX)

S8 Table Involvement of individual chromosomes in radiation induced inter-chromosome exchange events. Multicolor FISH data used for the generation of histogram plots presented in Fig 4 on the involvement of each of the homologous pair of autosomes and sex determining chromosomes in γ-rays induced inter-chromosome exchange events at different post-recovery times (2 hrs and 6 hrs) in human lymphocyte G0 PCCs are shown.
(DOCX)

## References

[1] Nugis VY, Filushkin IV, Chistopolskij AS. Retrospective dose estimation using the dicentric distribution in human peripheral lymphocytes. Applied radiation and isotopes: including data, instrumentation and methods for use in agriculture, industry and medicine. 2000;52(5):1139–44. Epub 2000/06/03. . 10836419 10.1016/s0969-8043(00)00060-9

[2] Sevan’kaev AV, Khvostunov IK, Mikhailova GF, Golub EV, Potetnya OI, Shepel NN, et al Novel data set for retrospective biodosimetry using both conventional and FISH chromosome analysis after high accidental overexposure. Applied radiation and isotopes: including data, instrumentation and methods for use in agriculture, industry and medicine. 2000;52(5):1149–52. Epub 2000/06/03. . 10836421 10.1016/s0969-8043(00)00062-2

[3] Sevan’kaev AV, Lloyd DC, Edwards AA, Khvostunov IK, Mikhailova GF, Golub EV, et al A cytogenetic follow-up of some highly irradiated victims of the Chernobyl accident. Radiation protection dosimetry. 2005;113(2):152–61. Epub 2004/12/02. 10.1093/rpd/nch435 . 15572397

[4] Natarajan AT, Ramalho AT, Vyas RC, Bernini LF, Tates AD, Ploem JS, et al Goiania radiation accident: results of initial dose estimation and follow up studies. Progress in clinical and biological research. 1991;372:145–53. Epub 1991/01/01. . 1956913

[5] Natarajan AT, Vyas RC, Darroudi F, Vermeulen S. Frequencies of X-ray-induced chromosome translocations in human peripheral lymphocytes as detected by in situ hybridization using chromosome-specific DNA libraries. International journal of radiation biology. 1992;61(2):199–203. Epub 1992/02/01. . 1351907 10.1080/09553009214550821

[6] Ramalho AT, Nascimento AC. The fate of chromosomal aberrations in 137Cs-exposed individuals in the Goiania radiation accident. Health physics. 1991;60(1):67–70. Epub 1991/01/01. . 1983985 10.1097/00004032-199101000-00010

[7] Lee JK, Han EA, Lee SS, Ha WH, Barquinero JF, Lee HR, et al Cytogenetic biodosimetry for Fukushima travelers after the nuclear power plant accident: no evidence of enhanced yield of dicentrics. Journal of radiation research. 2012;53(6):876–81. Epub 2012/08/04. 10.1093/jrr/rrs065 22859566 PMC3483860

[8] Suto Y. Review of Cytogenetic analysis of restoration workers for Fukushima Daiichi nuclear power station accident. Radiation protection dosimetry. 2016;171(1):61–3. Epub 2016/07/31. 10.1093/rpd/ncw187 . 27473701

[9] Suto Y, Hirai M, Akiyama M, Kobashi G, Itokawa M, Akashi M, et al Biodosimetry of restoration workers for the Tokyo Electric Power Company (TEPCO) Fukushima Daiichi nuclear power station accident. Health physics. 2013;105(4):366–73. Epub 2013/08/29. . 23982613 10.1097/HP.0b013e3182995e42

[10] Hill MA. Fishing for radiation quality: chromosome aberrations and the role of radiation track structure. Radiation protection dosimetry. 2015;166(1–4):295–301. Epub 2015/04/18. 10.1093/rpd/ncv151 . 25883310

[11] De Amicis A, De Sanctis S, Di Cristofaro S, Franchini V, Regalbuto E, Mammana G, et al Dose estimation using dicentric chromosome assay and cytokinesis block micronucleus assay: comparison between manual and automated scoring in triage mode. Health physics. 2014;106(6):787–97. Epub 2014/04/30. . 24776913 10.1097/HP.0000000000000097

[12] Gruel G, Gregoire E, Lecas S, Martin C, Roch-Lefevre S, Vaurijoux A, et al Biological dosimetry by automated dicentric scoring in a simulated emergency. Radiation research. 2013;179(5):557–69. Epub 2013/04/09. 10.1667/RR3196.1 . 23560627

[13] Liu J, Li Y, Wilkins R, Flegal F, Knoll JHM, Rogan PK. Accurate cytogenetic biodosimetry through automated dicentric chromosome curation and metaphase cell selection. F1000Research. 2017;6:1396 Epub 2017/10/14. 10.12688/f1000research.12226.1 29026522 PMC5583746

[14] Rogan PK, Li Y, Wilkins RC, Flegal FN, Knoll JH. Radiation Dose Estimation by Automated Cytogenetic Biodosimetry. Radiation protection dosimetry. 2016;172(1–3):207–17. Epub 2016/07/15. 10.1093/rpd/ncw161 . 27412514

[15] Romm H, Ainsbury E, Barnard S, Barrios L, Barquinero JF, Beinke C, et al Automatic scoring of dicentric chromosomes as a tool in large scale radiation accidents. Mutation research. 2013;756(1–2):174–83. Epub 2013/05/28. 10.1016/j.mrgentox.2013.05.013 . 23707243

[16] Schunck C, Johannes T, Varga D, Lorch T, Plesch A. New developments in automated cytogenetic imaging: unattended scoring of dicentric chromosomes, micronuclei, single cell gel electrophoresis, and fluorescence signals. Cytogenetic and genome research. 2004;104(1–4):383–9. Epub 2004/05/27. 10.1159/000077520 . 15162069

[17] Shirley B, Li Y, Knoll JHM, Rogan PK. Expedited Radiation Biodosimetry by Automated Dicentric Chromosome Identification (ADCI) and Dose Estimation. Journal of visualized experiments: JoVE. 2017;(127). Epub 2017/09/12. 10.3791/56245 28892030 PMC5619684

[18] Balajee AS, Smith T, Ryan T, Escalona M, Dainiak N. DEVELOPMENT OF A MINIATURIZED VERSION OF DICENTRIC CHROMOSOME ASSAY TOOL FOR RADIOLOGICAL TRIAGE. Radiation protection dosimetry. 2018;182(1):139–45. Epub 2018/09/25. 10.1093/rpd/ncy127 . 30247729

[19] Martin PR, Berdychevski RE, Subramanian U, Blakely WF, Prasanna PG. Sample Tracking in an Automated Cytogenetic Biodosimetry Laboratory for Radiation Mass Casualties. Radiation measurements. 2007;42(6–7):1119–24. Epub 2007/11/27. 10.1016/j.radmeas.2007.05.021 18037985 PMC2084199

[20] Blakely WF, Carr Z, Chu MC, Dayal-Drager R, Fujimoto K, Hopmeir M, et al WHO 1st consultation on the development of a global biodosimetry laboratories network for radiation emergencies (BioDoseNet). Radiation research. 2009;171(1):127–39. Epub 2009/01/14. 10.1667/RR1549.1 . 19138057

[21] Kulka U, Ainsbury L, Atkinson M, Barquinero JF, Barrios L, Beinke C, et al Realising the European Network of Biodosimetry (RENEB). Radiation protection dosimetry. 2012;151(4):621–5. Epub 2012/08/28. 10.1093/rpd/ncs157 . 22923244

[22] Wilkins RC, Romm H, Oestreicher U, Marro L, Yoshida MA, Suto Y, et al Biological Dosimetry by the Triage Dicentric Chromosome Assay—Further validation of International Networking. Radiation measurements. 2011;46(9):923–8. Epub 2011/09/29. 10.1016/j.radmeas.2011.03.012 21949482 PMC3176593

[23] Garcia O, Di Giorgio M, Vallerga MB, Radl A, Taja MR, Seoane A, et al Interlaboratory comparison of dicentric chromosome assay using electronically transmitted images. Radiation protection dosimetry. 2013;154(1):18–25. Epub 2012/08/08. 10.1093/rpd/ncs139 . 22869818

[24] Oestreicher U, Samaga D, Ainsbury E, Antunes AC, Baeyens A, Barrios L, et al RENEB intercomparisons applying the conventional Dicentric Chromosome Assay (DCA). International journal of radiation biology. 2017;93(1):20–9. Epub 2016/10/22. 10.1080/09553002.2016.1233370 . 27766931

[25] Romm H, Ainsbury EA, Barquinero JF, Barrios L, Beinke C, Cucu A, et al Web based scoring is useful for validation and harmonisation of scoring criteria within RENEB. International journal of radiation biology. 2017;93(1):110–7. Epub 2016/08/23. 10.1080/09553002.2016.1206228 . 27547893

[26] Sugarman SL, Livingston GK, Stricklin DL, Abbott MG, Wilkins RC, Romm H, et al The Internet’s role in a biodosimetric response to a radiation mass casualty event. Health physics. 2014;106(5 Suppl 2):S65–70. Epub 2014/03/29. . 24667387 10.1097/HP.0000000000000080

[27] Ainsbury EA, Livingston GK, Abbott MG, Moquet JE, Hone PA, Jenkins MS, et al Interlaboratory variation in scoring dicentric chromosomes in a case of partial-body x-ray exposure: implications for biodosimetry networking and cytogenetic "triage mode" scoring. Radiation research. 2009;172(6):746–52. Epub 2009/11/26. 10.1667/RR1934.1 . 19929421

[28] Di Giorgio M, Barquinero JF, Vallerga MB, Radl A, Taja MR, Seoane A, et al Biological dosimetry intercomparison exercise: an evaluation of triage and routine mode results by robust methods. Radiation research. 2011;175(5):638–49. Epub 2011/02/11. 10.1667/RR2425.1 . 21306200

[29] Romm H, Ainsbury E, Bajinskis A, Barnard S, Barquinero JF, Barrios L, et al Web-based scoring of the dicentric assay, a collaborative biodosimetric scoring strategy for population triage in large scale radiation accidents. Radiation and environmental biophysics. 2014;53(2):241–54. Epub 2014/02/22. 10.1007/s00411-014-0519-8 . 24557539

[30] Pantelias GE, Maillie HD. A simple method for premature chromosome condensation induction in primary human and rodent cells using polyethylene glycol. Somatic cell genetics. 1983;9(5):533–47. Epub 1983/09/01. . 6623312 10.1007/BF01574257

[31] Pantelias GE, Maillie HD. The use of peripheral blood mononuclear cell prematurely condensed chromosomes for biological dosimetry. Radiation research. 1984;99(1):140–50. Epub 1984/07/01. . 6539927

[32] Pantelias GE, Maillie HD. Direct analysis of radiation-induced chromosome fragments and rings in unstimulated human peripheral blood lymphocytes by means of the premature chromosome condensation technique. Mutation research. 1985;149(1):67–72. Epub 1985/03/01. . 3974623 10.1016/0027-5107(85)90010-7

[33] Darroudi F, Natarajan AT, Bentvelzen PA, Heidt PJ, Van Rotterdam A, Zoetelief J, et al Detection of total- and partial-body irradiation in a monkey model: a comparative study of chromosomal aberration, micronucleus and premature chromosome condensation assays. International journal of radiation biology. 1998;74(2):207–15. Epub 1998/08/26. . 9712549 10.1080/095530098141582

[34] Pantelias GE, Iliakis GE, Sambani CD, Politis G. Biological dosimetry of absorbed radiation by C-banding of interphase chromosomes in peripheral blood lymphocytes. International journal of radiation biology. 1993;63(3):349–54. Epub 1993/03/01. . 8095285 10.1080/09553009314550461

[35] Karachristou I, Karakosta M, Pantelias A, Hatzi VI, Karaiskos P, Dimitriou P, et al Triage biodosimetry using centromeric/telomeric PNA probes and Giemsa staining to score dicentrics or excess fragments in non-stimulated lymphocyte prematurely condensed chromosomes. Mutation research Genetic toxicology and environmental mutagenesis. 2015;793:107–14. Epub 2015/11/02. 10.1016/j.mrgentox.2015.06.013 . 26520380

[36] M’Kacher R, Maalouf EE, Ricoul M, Heidingsfelder L, Laplagne E, Cuceu C, et al New tool for biological dosimetry: reevaluation and automation of the gold standard method following telomere and centromere staining. Mutation research. 2014;770:45–53. Epub 2015/03/17. 10.1016/j.mrfmmm.2014.09.007 . 25771869

[37] Suto Y, Gotoh T, Noda T, Akiyama M, Owaki M, Darroudi F, et al Assessing the applicability of FISH-based prematurely condensed dicentric chromosome assay in triage biodosimetry. Health physics. 2015;108(3):371–6. Epub 2015/01/30. 10.1097/HP.0000000000000182 . 25627950

[38] Terzoudi GI, Pantelias G, Darroudi F, Barszczewska K, Buraczewska I, Depuydt J, et al Dose assessment intercomparisons within the RENEB network using G0-lymphocyte prematurely condensed chromosomes (PCC assay). International journal of radiation biology. 2017;93(1):48–57. Epub 2016/11/05. 10.1080/09553002.2016.1234725 27813725 PMC5495998

[39] Livingston GK, Escalona M, Foster A, Balajee AS. Persistent in vivo cytogenetic effects of radioiodine therapy: a 21-year follow-up study using multicolor FISH. Journal of radiation research. 2018;59(1):10–7. Epub 2017/10/17. 10.1093/jrr/rrx049 29036595 PMC5778502

[40] Smith T, Escalona M, Ryan T, Livingston GK, Sanders JT, Balajee AS. Extension of lymphocyte viability for radiation biodosimetry: Potential implications for radiological/nuclear mass casualty incidents. Journal of cellular biochemistry. 2018 Epub 2018/12/12. 10.1002/jcb.28150 . 30536664

[41] M’Kacher R, El Maalouf E, Terzoudi G, Ricoul M, Heidingsfelder L, Karachristou I, et al Detection and automated scoring of dicentric chromosomes in nonstimulated lymphocyte prematurely condensed chromosomes after telomere and centromere staining. International journal of radiation oncology, biology, physics. 2015;91(3):640–9. Epub 2015/01/18. 10.1016/j.ijrobp.2014.10.048 . 25596111

[42] Lamadrid Boada AI, Romero Aguilera I, Terzoudi GI, Gonzalez Mesa JE, Pantelias G, Garcia O. Rapid assessment of high-dose radiation exposures through scoring of cell-fusion-induced premature chromosome condensation and ring chromosomes. Mutation research. 2013;757(1):45–51. Epub 2013/07/16. 10.1016/j.mrgentox.2013.06.021 . 23850809

[43] Okayasu R, Pantelias GE, Iliakis G. Increased frequency of formation of interphase ring-chromosomes in radiosensitive irs-1 cells exposed to X-rays. Mutation research. 1993;294(3):199–206. Epub 1993/10/01. . 7692259 10.1016/0921-8777(93)90002-x

[44] Sipi P, Lindholm C, Salomaa S. Kinetics of formation of exchanges and rejoining of breaks in human G0 and G2 lymphocytes after low-LET radiation. International journal of radiation biology. 2000;76(6):823–30. Epub 2000/07/21. . 10902737 10.1080/09553000050028986

[45] Terzoudi GI, Pantelias GE. Conversion of DNA damage into chromosome damage in response to cell cycle regulation of chromatin condensation after irradiation. Mutagenesis. 1997;12(4):271–6. Epub 1997/07/01. . 9237773 10.1093/mutage/12.4.271

[46] Pantelias A, Terzoudi GI. Development of an automatable micro-PCC biodosimetry assay for rapid individualized risk assessment in large-scale radiological emergencies. Mutation research. 2018;836(Pt A):65–71. Epub 2018/11/06. 10.1016/j.mrgentox.2018.05.013 . 30389164 PMC6486952

[47] Durante M, Kawata T, Nakano T, Yamada S, Tsujii H. Biodosimetry of heavy ions by interphase chromosome painting. Advances in space research: the official journal of the Committee on Space Research (COSPAR). 1998;22(12):1653–62. Epub 2001/09/07. . 11542409 10.1016/s0273-1177(99)00030-7

[48] Foster HA, Estrada-Girona G, Themis M, Garimberti E, Hill MA, Bridger JM, et al Relative proximity of chromosome territories influences chromosome exchange partners in radiation-induced chromosome rearrangements in primary human bronchial epithelial cells. Mutation research. 2013;756(1–2):66–77. Epub 2013/06/26. 10.1016/j.mrgentox.2013.06.003 . 23791770

[49] Greinert R, Detzler E, Volkmer B, Harder D. Kinetics of the formation of chromosome aberrations in X-irradiated human lymphocytes: analysis by premature chromosome condensation with delayed fusion. Radiation research. 1995;144(2):190–7. Epub 1995/11/01. . 7480645

[50] Bauchinger M, Schmid E, Zitzelsberger H, Braselmann H, Nahrstedt U. Radiation-induced chromosome aberrations analysed by two-colour fluorescence in situ hybridization with composite whole chromosome-specific DNA probes and a pancentromeric DNA probe. International journal of radiation biology. 1993;64(2):179–84. Epub 1993/08/01. . 8103541 10.1080/09553009314551271

[51] Bauchinger M, Schmid E. LET dependence of yield ratios of radiation-induced intra- and interchromosomal aberrations in human lymphocytes. International journal of radiation biology. 1998;74(1):17–25. Epub 1998/08/04. . 9687971 10.1080/095530098141681

[52] Natarajan AT, Balajee AS, Boei JJ, Chatterjee S, Darroudi F, Grigorova M, et al Recent developments in the assessment of chromosomal damage. International journal of radiation biology. 1994;66(5):615–23. Epub 1994/11/01. . 7983455 10.1080/09553009414551711

[53] Natarajan AT, Balajee AS, Boei JJ, Darroudi F, Dominguez I, Hande MP, et al Mechanisms of induction of chromosomal aberrations and their detection by fluorescence in situ hybridization. Mutation research. 1996;372(2):247–58. Epub 1996/12/01. . 9015143 10.1016/s0027-5107(96)00144-3

[54] Holmberg M, Gumauskas E. The role of short-lived DNA lesions in the production of chromosome-exchange aberrations. Mutation research. 1986;160(3):221–9. Epub 1986/05/01. . 3960035 10.1016/0027-5107(86)90131-4

[55] Natarajan AT, van Zeeland AA, Zwanenburg TS. Influence of inhibitors of poly(ADP-ribose) polymerase on DNA repair, chromosomal alterations, and mutations. Princess Takamatsu symposia. 1983;13:227–42. Epub 1983/01/01. . 6317638

[56] Preston RJ. The effect of cytosine arabinoside on the frequency of X-ray-induced chromosome aberrations in normal human leukocytes. Mutation research. 1980;69(1):71–9. Epub 1980/01/01. . 7360145 10.1016/0027-5107(80)90177-3

[57] Manti L, Durante M, Grossi G, Ortenzia O, Pugliese M, Scampoli P, et al Measurements of metaphase and interphase chromosome aberrations transmitted through early cell replication rounds in human lymphocytes exposed to low-LET protons and high-LET 12C ions. Mutation research. 2006;596(1–2):151–65. Epub 2006/02/08. 10.1016/j.mrfmmm.2005.12.010 . 16460768

[58] Muhlmann-Diaz MC, Bedford JS. Breakage of human chromosomes 4, 19 and Y in G0 cells immediately after exposure to gamma-rays. International journal of radiation biology. 1994;65(2):165–73. Epub 1994/02/01. . 7907114 10.1080/09553009414550201

[59] Johannes C, Chudoba I, Obe G. Analysis of X-ray-induced aberrations in human chromosome 5 using high-resolution multicolour banding FISH (mBAND). Chromosome research: an international journal on the molecular, supramolecular and evolutionary aspects of chromosome biology. 1999;7(8):625–33. Epub 2000/01/11. . 10628663 10.1023/a:1009284018942

