# Supplementary material for: Use of human lymphocyte G0 PCCs to detect intra- and inter-chromosomal aberrations for early radiation biodosimetry and retrospective assessment of radiation-induced effects
Source: PLoS One. 2019 May 6;14(5):e0216081. doi: 10.1371/journal.pone.0216081 (PMC6502328; doi:10.1371/journal.pone.0216081)
Supplement: S1 Table — Distribution of dicentric chromosomes observed in a cell to cell basis and the range of total chromosome objected observed including excess fragments for different doses are shown. (DOCX) [file pone.0216081.s001.docx]

**Supplemental Data**

| **S1.Table. Detection of dicentric chromosomes in γ-rays irradiated human lymphocyte** | | | |
| --- | --- | --- | --- |
| **G0 PCCs by centromere/telomere FISH (Raw Data)**   \| Radiation Dose \| 0Gy \| 1Gy \| 2Gy \| 4Gy \| 6Gy \| \| --- \| --- \| --- \| --- \| --- \| --- \| \| average object number* \| 46 \| 46 \| 47.5 \| 48.8 \| 52.6 \| \| range \| 44-47 \| 45-48 \| 45-50 \| 46-52 \| 48-58 \| \| cells w/ 0 dicentrics \| 50 \| 47 \| 45 \| 12 \| 6 \| \| cells w/ 1 dicentric \| 0 \| 3 \| 3 \| 20 \| 8 \| \| cells w/ 2 dicentrics \| 0 \| 0 \| 2 \| 10 \| 10 \| \| cells w/ 3 dicentrics \| 0 \| 0 \| 0 \| 6 \| 14 \| \| cells w/ 4 dicentrics \| 0 \| 0 \| 0 \| 2 \| 8 \| \| cells w/ 5 dicentrics \| 0 \| 0 \| 0 \| 0 \| 3 \| \| cells w/ 6 dicentrics \| 0 \| 0 \| 0 \| 0 \| 0 \| \| cells w/ 7 dicentrics \| 0 \| 0 \| 0 \| 0 \| 1 \| \| Total cells analyzed \| 50 \| 50 \| 50 \| 50 \| 50 \| \| Total Dicentrics \| 0 \| 3 \| 7 \| 66 \| 124 \| \| Frequency/Cell \| 0 \| 0.06 \| 0.14 \| 1.32 \| 2.48 \| |  |  |  |

* Indicates the number of chromosome objects. Excess fragments (> 46±1) indicate chromosome breakage
