# Supplementary material for: Use of human lymphocyte G0 PCCs to detect intra- and inter-chromosomal aberrations for early radiation biodosimetry and retrospective assessment of radiation-induced effects
Source: PLoS One. 2019 May 6;14(5):e0216081. doi: 10.1371/journal.pone.0216081 (PMC6502328; doi:10.1371/journal.pone.0216081)
Supplement: S2 Table — Distribution of chromosome exchange events observed for varying radiation doses are shown. (DOCX) [file pone.0216081.s002.docx]

**S2 Table. Detection of γ-rays induced inter-chromosome exchange events detected by whole chromosome specific DNA cocktail probe (Chr.1, 2 and 4; Raw data)**

| **Exchange events/Cell** | **0 Gy** | **1 Gy** | **2 Gy** | **4 Gy** | **6 Gy** |
| --- | --- | --- | --- | --- | --- |
| 0 | 99 | 93 | 58 | 36 | 12 |
| 1 | 1 | 3 | 5 | 26 | 10 |
| 2 | 0 | 4 | 3 | 20 | 13 |
| 3 | 0 | 0 | 1 | 8 | 9 |
| 4 | 0 | 0 | 0 | 7 | 9 |
| 5 | 0 | 0 | 0 | 2 | 8 |
| 6 | 0 | 0 | 0 | 0 | 12 |
| 7 | 0 | 0 | 0 | 1 | 6 |
| 8 | 0 | 0 | 0 | 0 | 2 |
| 9 | 0 | 0 | 0 | 0 | 2 |
| 10 | 0 | 0 | 0 | 0 | 2 |
| **Cells analyzed** | **100** | **100** | **67** | **100** | **85** |
| **Total color junctions** | **1** | **11** | **14** | **135** | **307** |
| **Frequency/Cell** | **0.01** | **0.11** | **0.21** | **1.35** | **3.61** |
|  |  |  |  |  |  |
