# Supplementary material for: Use of human lymphocyte G0 PCCs to detect intra- and inter-chromosomal aberrations for early radiation biodosimetry and retrospective assessment of radiation-induced effects
Source: PLoS One. 2019 May 6;14(5):e0216081. doi: 10.1371/journal.pone.0216081 (PMC6502328; doi:10.1371/journal.pone.0216081)
Supplement: S3 Table — Cellular distribution of chromosome exchange events observed for different doses of γ-rays is shown. (DOCX) [file pone.0216081.s003.docx]

**S3 Table. Detection of γ-rays induced inter-chromosome exchange events detected by multicolor FISH in human G0 lymphocyte PCCs prepared 2 hrs after exposure (Raw data)**

| **Exchange events/cell** | **0Gy** | **1Gy** | **2Gy** | **4Gy** | **6Gy** |
| --- | --- | --- | --- | --- | --- |
| 0 | 30 | 20 | 18 | 9 | 0 |
| 1 | 0 | 5 | 5 | 5 | 0 |
| 2 | 0 | 4 | 4 | 7 | 0 |
| 3 | 0 | 1 | 1 | 4 | 1 |
| 4 | 0 | 0 | 2 | 2 | 4 |
| 5 | 0 | 0 | 0 | 2 | 11 |
| 6 | 0 | 0 | 0 | 1 | 4 |
| 7 | 0 | 0 | 0 | 0 | 1 |
| 8 | 0 | 0 | 0 | 0 | 1 |
| 9 | 0 | 0 | 0 | 0 | 3 |
| 10 | 0 | 0 | 0 | 0 | 0 |
| **Total exchange events** | **0** | **16** | **24** | **55** | **140** |
| **Total cells analyzed** | **30** | **30** | **30** | **30** | **25** |
| **Frequency/Cell** | **0** | **0.53** | **0.80** | **1.83** | **5.60** |
