# Supplementary material for: Use of human lymphocyte G0 PCCs to detect intra- and inter-chromosomal aberrations for early radiation biodosimetry and retrospective assessment of radiation-induced effects
Source: PLoS One. 2019 May 6;14(5):e0216081. doi: 10.1371/journal.pone.0216081 (PMC6502328; doi:10.1371/journal.pone.0216081)
Supplement: S4 Table — Cells with varying number of inter-chromosome exchange events (0–16) observed after exposure to varying radiation doses are shown. (DOCX) [file pone.0216081.s004.docx]

**S4 Table. Detection of γ-rays induced inter-chromosome exchange events detected by multicolor FISH in human G0 lymphocyte PCCs prepared 6 hrs after exposure (Raw data)**

| **Exchange events/cell** | **0Gy** | **1Gy** | **2Gy** | **4Gy** | **6Gy** |
| --- | --- | --- | --- | --- | --- |
| 0 | 30 | 19 | 16 | 2 | 0 |
| 1 | 0 | 5 | 1 | 8 | 0 |
| 2 | 0 | 5 | 8 | 3 | 0 |
| 3 | 0 | 1 | 1 | 2 | 1 |
| 4 | 0 | 0 | 3 | 1 | 1 |
| 5 | 0 | 0 | 0 | 2 | 1 |
| 6 | 0 | 0 | 1 | 3 | 2 |
| 7 | 0 | 0 | 0 | 5 | 1 |
| 8 | 0 | 0 | 0 | 2 | 5 |
| 9 | 0 | 0 | 0 | 1 | 4 |
| 10 | 0 | 0 | 0 | 0 | 2 |
| 11 | 0 | 0 | 0 | 1 | 2 |
| 12 | 0 | 0 | 0 | 0 | 2 |
| 13 | 0 | 0 | 0 | 0 | 1 |
| 14 | 0 | 0 | 0 | 0 | 2 |
| 15 | 0 | 0 | 0 | 0 | 0 |
| 16 | 0 | 0 | 0 | 0 | 1 |
| 17 | 0 | 0 | 0 | 0 | 0 |
| **Total exchange events** | **0** | **18** | **38** | **123** | **230** |
| **Total cells analyzed** | **30** | **30** | **30** | **30** | **25** |
| **Frequency/Cell** | **0.00** | **0.60** | **1.27** | **4.10** | **9.20** |
