# Supplementary material for: Use of human lymphocyte G0 PCCs to detect intra- and inter-chromosomal aberrations for early radiation biodosimetry and retrospective assessment of radiation-induced effects
Source: PLoS One. 2019 May 6;14(5):e0216081. doi: 10.1371/journal.pone.0216081 (PMC6502328; doi:10.1371/journal.pone.0216081)
Supplement: S5 Table — Cellular distribution of chromosome exchange events induced by X-rays is shown. (DOCX) [file pone.0216081.s005.docx]

**S5 Table. Detection of X-rays induced inter-chromosome exchange events in prematurely condensed human chromosomes using whole chromosome specific DNA cocktail probe (Chr.1, 2 and 4; Raw data)**

| **Exchange events/cell** | **0Gy** | **2Gy** | **4Gy** |
| --- | --- | --- | --- |
| 0 | 100 | 46 | 31 |
| 1 | 0 | 13 | 11 |
| 2 | 0 | 5 | 12 |
| 3 | 0 | 1 | 6 |
| **Total exchange events** | **0** | **26** | **53** |
| **Total cells analyzed** | **100** | **65** | **60** |
| **Frequency/Cell** | **0.00** | **0.40** | **0.85** |
