# Supplementary material for: Use of human lymphocyte G0 PCCs to detect intra- and inter-chromosomal aberrations for early radiation biodosimetry and retrospective assessment of radiation-induced effects
Source: PLoS One. 2019 May 6;14(5):e0216081. doi: 10.1371/journal.pone.0216081 (PMC6502328; doi:10.1371/journal.pone.0216081)
Supplement: S6 Table — Cellular distribution of chromosome exchange events are shown for various doses of X-rays. (DOCX) [file pone.0216081.s006.docx]

**S6 Table. Detection of X-rays induced inter-chromosome exchange events in prematurely condensed human chromosomes using multicolor FISH; Raw data)**

| **Exchange events/cell** | **0 Gy** | **2 Gy** | **4 Gy** |
| --- | --- | --- | --- |
| 0 | 49 | 21 | 8 |
| 1 | 1 | 1 | 2 |
| 2 | 0 | 2 | 3 |
| 3 | 0 | 1 | 10 |
| 4 | 0 | 2 | 4 |
| 5 | 0 | 1 | 1 |
| **Total number of Exchanges** | **1** | **21** | **59** |
| **Total number of cells analyzed** | **50** | **28** | **28** |
| **Frequency/Cell** | **0.02** | **0.75** | **2.10** |
