# Supplementary material for: Use of human lymphocyte G0 PCCs to detect intra- and inter-chromosomal aberrations for early radiation biodosimetry and retrospective assessment of radiation-induced effects
Source: PLoS One. 2019 May 6;14(5):e0216081. doi: 10.1371/journal.pone.0216081 (PMC6502328; doi:10.1371/journal.pone.0216081)
Supplement: S7 Table — Data used for the generation of histogram plots presented in Fig 5D and 5E are shown. (DOCX) [file pone.0216081.s007.docx]

**S7 Table. Analysis of γ-rays induced intra-chromosomal exchanges detected by the mBAND technique on chromosome 5. Raw data used for the generation of histograms presented in Fig. 5 D and E are shown below:**

|  | **0Gy** | **2Gy** | **4Gy** | **6Gy** |
| --- | --- | --- | --- | --- |
| **Cells with two normal chromosome 5** | **99** | **69** | **43** | **28** |
| **One of the homologs with one break** | **0** | **9** | **15** | **18** |
| band p14-15 |  | 2 (fr) |  | 1(fr) |
| band p13 |  |  |  |  |
| band p11.2-p12 |  | 3 (fr) | 2 (fr) | 5(fr) 1(p arm deleted) |
| band q12 |  |  |  | 1(fr) 1(p arm deleted) |
| band q13 |  |  |  |  |
| band q14 |  |  | 5 (fr) | 2(fr) |
| band q15-q22 |  | 2 (fr) |  |  |
| band q23 |  | 1 (fr) | 2 (fr) | 1(fr) |
| band q31 |  |  | 1 (fr) | 1(fr) |
| band q32 |  |  |  |  |
| band q33 |  | 1 (fr) | 4 (fr) | 3(fr) |
| band q35 |  |  | 1 (fr) | 2(fr) |
| **One of the homologs with two breaks** | **0** | **1** | **2** | **4** |
| band p14-15 |  |  |  |  |
| band p13 |  |  |  |  |
| band p11.2-p12 |  | 1 (trans) | 1 (fr) | 1(fr)* 1(fr)' 1(fr)" |
| band q12 |  |  |  |  |
| band q13 |  |  |  |  |
| band q14 |  |  | 1 (fr) |  |
| band q15-q22 |  |  |  | 1(fr)~ |
| band q23 |  |  |  |  |
| band q31 |  |  |  | 1(fr)' |
| band q32 |  |  | 1 (fr) |  |
| band q33 |  | 1(fr) |  | 1(fr)" 1(fr)~ |
| band q35 |  |  | 1 (fr) | 1(fr)* |
| **One of the homologs with four breaks** | **0** | **0** | **0** | **1** |
| band p14-15 |  |  |  |  |
| band p13 |  |  |  | 1(fr) |
| band p11.2-p12 |  |  |  |  |
| band q12 |  |  |  |  |
| band q13 |  |  |  |  |
| band q14 |  |  |  |  |
| band q15-q22 |  |  |  |  |
| band q23 |  |  |  |  |
| band q31 |  |  |  | 1(fr) |
| band q32 |  |  |  |  |
| band q33 |  |  |  | 1(fr) |
| band q35 |  |  |  | 1(fr) |
| **Both homologs with one break** | **0** | **0** | **1** | **5** |
| band p14-15 |  |  |  | 1(fr) |
| band p13 |  |  |  | 1(fr) |
| band p11.2-p12 |  |  | 1(fr) | 1(fr) 1(fr) |
| band q12 |  |  |  | 1(fr) |
| band q13 |  |  |  |  |
| band q14 |  |  |  | 1(fr) |
| band q15-q22 |  |  |  | 1(fr) |
| band q23 |  |  | 1(fr) | 1(fr) |
| band q31 |  |  |  |  |
| band q32 |  |  |  |  |
| band q33 |  |  |  | 1(fr) |
| band q35 |  |  |  | 1(fr) |
| **One homolog with one and the other with two breaks** | **0** | **0** | **1** | **2** |
| band p14-15 |  |  |  | 1(fr)* 1(fr)' |
| band p13 |  |  |  |  |
| band p11.2-p12 |  |  |  | 1(trans)* 1(fr)' |
| band q12 |  |  |  |  |
| band q13 |  |  |  |  |
| band q14 |  |  |  | 1(fr)* 1(fr)' |
| band q15-q22 |  |  | 1(fr) |  |
| band q23 |  |  | 1(fr)/1(fr) |  |
| band q31 |  |  |  |  |
| band q32 |  |  |  |  |
| band q33 |  |  |  |  |
| band q35 |  |  |  |  |
| **extra chromosome material present** | **0** | **0** | **1** | **0** |
| band p11.2-p12 |  |  | 1(fr) |  |
| **Total cells scored** | **100** | **80** | **65** | **60** |
| **Total breaks detected** | **0** | **10** | **24** | **45** |
| **Translocations** | **0** | **1** | **3** | **1** |
| **Total aberrations** | **0** | **11** | **27** | **46** |
| **Aberration Frequency/Cell** | **0** | **0.13** | **0.41** | **0.76** |

***, ~, ' and "- Breaks observed at different locations on the same homologous chromosome 5.**
