# Supplementary material for: Use of human lymphocyte G0 PCCs to detect intra- and inter-chromosomal aberrations for early radiation biodosimetry and retrospective assessment of radiation-induced effects
Source: PLoS One. 2019 May 6;14(5):e0216081. doi: 10.1371/journal.pone.0216081 (PMC6502328; doi:10.1371/journal.pone.0216081)
Supplement: S8 Table — Multicolor FISH data used for the generation of histogram plots presented in Fig 4 on the involvement of each of the homologous pair of autosomes and sex determining chromosomes in γ-rays induced inter-chromosome exchange events at different post-recovery times (2 hrs and 6 hrs) in human lymphocyte G0 PCCs are shown. (DOCX) [file pone.0216081.s008.docx]

**S8 Table. Involvement of individual chromosomes in radiation induced inter-chromosome exchange events.**

Multicolor FISH data used for the generation of histogram plots presented in Fig. 4 on the involvement of each of the homologous pair of autosomes and sex determining chromosomes in γ-rays induced inter-chromosome exchange events at different post-recovery times (2 hrs and 6 hrs) in human lymphocyte G0 PCCs are shown below:

|  | **0 Gy** | **1 Gy** | **1 Gy** | **2 Gy** | **2 Gy** | **4 Gy** | **4Gy** | **6 Gy** | **6 Gy** |
| --- | --- | --- | --- | --- | --- | --- | --- | --- | --- |
| **Chromosomes** | **6 hr** | **2hr** | **6hr** | **2hr** | **6hr** | **2hr** | **6hr** | **2hr** | **6hr** |
| 1 | 0 | **2** | **1** | **0** | **1** | **8** | **21** | **16** | **24** |
| 2 | 0 | **2** | **2** | **6** | **1** | **5** | **17** | **24** | **30** |
| 3 | 0 | **4** | **2** | **8** | **7** | **7** | **16** | **11** | **32** |
| 4 | 0 | **4** | **2** | **3** | **3** | **9** | **10** | **28** | **30** |
| 5 | 0 | **0** | **0** | **1** | **5** | **4** | **14** | **10** | **34** |
| 6 | 0 | **0** | **3** | **3** | **9** | **4** | **9** | **9** | **23** |
| 7 | 0 | **4** | **2** | **6** | **4** | **7** | **18** | **16** | **24** |
| 8 | 0 | **1** | **5** | **3** | **5** | **9** | **7** | **10** | **21** |
| 9 | 0 | **1** | **4** | **2** | **1** | **4** | **10** | **11** | **22** |
| 10 | 0 | **0** | **2** | **2** | **3** | **6** | **13** | **19** | **26** |
| 11 | 0 | **2** | **0** | **1** | **1** | **4** | **13** | **12** | **19** |
| 12 | 0 | **0** | **0** | **2** | **8** | **7** | **17** | **14** | **17** |
| 13 | 0 | **0** | **1** | **1** | **3** | **6** | **4** | **14** | **15** |
| 14 | 0 | **3** | **2** | **2** | **3** | **6** | **11** | **13** | **19** |
| 15 | 0 | **2** | **3** | **2** | **6** | **5** | **6** | **13** | **17** |
| 16 | 0 | **3** | **0** | **2** | **2** | **4** | **10** | **12** | **20** |
| 17 | 0 | **1** | **1** | **2** | **0** | **2** | **9** | **9** | **16** |
| 18 | 0 | **2** | **4** | **0** | **6** | **2** | **9** | **5** | **15** |
| 19 | 0 | **0** | **0** | **0** | **2** | **0** | **1** | **6** | **7** |
| 20 | 0 | **0** | **0** | **0** | **3** | **4** | **8** | **6** | **8** |
| 21 | 0 | **1** | **0** | **0** | **0** | **2** | **5** | **5** | **9** |
| 22 | 0 | **0** | **2** | **0** | **2** | **2** | **6** | **2** | **7** |
| X | 0 | **0** | **0** | **2** | **1** | **2** | **10** | **12** | **17** |
| Y | 0 | **0** | **0** | **0** | **0** | **1** | **2** | **3** | **8** |
| **Total exchanges** | **0** | **16** | **18** | **24** | **38** | **55** | **123** | **140** | **230** |
| **Total cells analyzed** | **30** | **30** | **30** | **30** | **30** | **30** | **30** | **25** | **25** |
